# The polycomb group protein EZH2 is a novel therapeutic target in tongue cancer

**DOI:** 10.18632/oncotarget.1503

**Published:** 2013-12-07

**Authors:** Zhongwu Li, Yanling Wang, Jing Qiu, Qiang Li, Chunping Yuan, Wei Zhang, Dongmiao Wang, Jinhai Ye, Hongbin Jiang, Jianrong Yang, Jie Cheng

**Affiliations:** ^1^ Head Neck Cancer Center, Institute of Stomatology, Affiliated Stomatological Hospital, Nanjing Medical University, Jiangsu, China PRC; ^2^ Department of Oral and Maxillofacial Surgery, Affiliated Stomatological Hospital, Nanjing Medical University, Jiangsu, China PRC

**Keywords:** tongue squamous cell carcinoma, polycomb complex, EZH2, DZNep

## Abstract

EZH2, a core member of the Polycomb Repressor Complex 2 (PRC2), mediates transcriptional silencing by catalyzing the trimethylation of histone 3 lysine 27 (H3K27), which plays key roles in cancer initiation and progression. Here, we investigated the expression pattern and biological roles of EZH2 in tongue tumorigenesis by loss-of-function assays using small interference RNA and EZH2 inhibitor DZNep. Also we determined the therapeutic efficiency of DZNep against tongue cancer in vivo. We found that aberrantly overexpressed EZH2 was associated with pathological grade, cervical nodes metastasis and Ki-67 expression in tongue cancers. Elevated EZH2 correlated with shorter overall survival and showed significant and independent prognostic importance in patients with tongue cancer. Both genetic and pharmacological depletion of EZH2 inhibited cell proliferation, migration, invasion and colony formation and decreased CD44+ subpopulation probably in part through modulating p16, p21 and E-caherin. Moreover, DZNep enhanced the anticancer effects of 5-Fluorouracil. Furthermore, intratumoral EZH2 inhibition induced by DZNep intraperitoneal administration significantly attenuated tumor growth in a tongue cancer xenograft model. Taken together, our results indicate that EZH2 serves as a key driver with multiple oncogenic functions during tongue tumorigenesis and a new biomarker for tongue cancer diagnosis and prognostic prediction. These findings open up possibilities for therapeutic intervention against EZH2 in tongue cancer.

## INTRODUCTION

Oral cancer is the sixth most common cancers worldwide with more than 70% of cases occurring in developing countries, accounting for approximately 3% of all malignancies in both sexes [1]. The major risks for this malignancy include human papillomavirus (HPV) infection, smoking and heavy alcohol consumption [2]. The overweighing majority of oral cancer is diagnosed as squamous cell carcinoma (SCC) and mostly arises from tongue. Despite tremendous advancement in multimodal therapies for oral cancer including surgery, chemotherapy and radiotherapy over the past decades, the 5-year survival rate of oral SCC has not increased too much. Local relapse and cervical lymph node metastasis are the most prevalent factor affecting patients' survival [3]. Notably, some small or early oral carcinoma lesions may have occult nodal metastasis and behave aggressively at their initial stages. Many patients are diagnosed at an advanced stage in their initial clinical visits and died without successful treatments. Although many oncogenes and tumor suppressors have been identified as key players underlying oral tumorigenesis, however, no optimal and commonly-accepted biomarkers have been established to facilitate the comprehensive management of patients, for example timely diagnosis, treatment selection and prognostic prediction [4]. These facts underscore the aggressive nature of oral cancer and therapeutic challenge for clinicians. Therefore, the identifications of the new biomarkers and therapeutic targets for oral SCC especially the tongue SCC (TSCC) are paramount and urgent to optimize diagnosis and treatment strategies for this lethal disease.

The enhancer of zest homolog (EZH2) is a core catalytic subunit of the polycomb repressive complex 2 (PRC2), which epigenetically regulates genes by specifically trimethylating nucleosomal histone H3 at lysine 27(H3K27me3) [5]. Gene silencing mediated by EZH2-induced H3K27me3 has been involved in diverse fundamental cellular processes, such as cell fate decision, cell cycle progression, apoptosis and senescence, stem cell maintenance and cancer development [6]. Previous reports have established that EZH2 is aberrantly overexpressed in a wide range of cancer types including breast, prostate, lung cancer and so forth [7-9]. EZH2 overexpression often correlated with advanced stages and poor prognosis in these cancers. Enforced expression of EZH2 increased cancer cell proliferation, epithelial-mesenchymal transition, metastatic spreading and other oncogenic properties, whereas its depletion inhibited cell proliferation, migration and invasion and induced cell apoptosis and senescence both in vitro and in vivo [10-12]. Furthermore, accumulated evidence indicates that a myriad of direct or indirect target genes including E-cadherin, INK-ARP(p14,p16) are partially responsible for the essential roles of EZH2 in various cancers [13, 14]. These findings have established that EZH2 functions as an important oncogenic biomarker for cancer initiation and progression, thus leading to the hypothesis that blocking EZH2 expression/activity and its downstream signaling cascade may represent a promising strategy for novel anticancer treatment. Indeed, several reports have shown that genetic silencing and pharmacologic inhibition of EZH2 induced cell apoptosis, inhibited cell invasion and tumor angiogenesis, ultimately suppressed cancer growth and progression [15, 16]. More importantly, given the advantages of specific chemical compounds including convenient to use and reversible nature of epigenetic modifications behind carcinogenesis, administration of small molecules targeting EZH2 seems to be a plausible and appealing way as a novel anti-cancer strategy [17].

3-Deazaneplanocin A (DZNep) is the cyclopentanyl analog of 3-deazaadenosine that potently inhibits the activity of S-adenosylhomocyteine (AdoHcy) hydrolase, resulting in cellular accumulation of AdoHcy which in turn represses the S-adenosyl-L-methionine-dependent histone lysine methyltransferase activities [18]. Several lines of evidence indicates that DZNep appears to be a unique chromatin-remodeling compound that induces degradation of cellular PRC2 proteins including EZH2 and concomitant removal of H3K27me3 mark, and reactivates the epigenetically silenced targets [19]. Disruption of EZH2 by DZNep induced apoptosis, inhibited cell invasion and enhanced chemotherapeutic sensitivity, but not normal and untransformed cells at tumor-inhibiting doses [20]. Moreover, DZNep-induced inhibition of EZH2 dramatically diminished the number and self-renewal capacity of cancer cells with tumor-initiating properties and significantly decreased tumor xenograft growth and improved survival [21, 22]. These findings suggested DZNep may be a promising therapeutic agent for cancer treatment through multiple characterized and unknown mechanisms.

Accumulating evidence has indicated that EZH2 serves as an essential oncogenic driver during the initiation and progression of head neck cancers. Overexpression of EZH2 significantly correlated with tumor size, cervical lymph node metastasis, clinical stage and poor prognosis, and served as an independent prognostic indicator for patients with head neck cancer [23-25]. However, the in-depth investigations into the expression pattern of EZH2 and associated molecular mechanisms underlying TSCC development has been in infancy and remains to be further clarified. Additionally, the therapeutic efficiency of EZH2-targeting compounds like DZNep as a new option for TSCC treatment remains largely unknown. Therefore, in the present study, we first examined the EZH2 expression and its clinicopathological significance in tongue cancer cell lines and clinical samples. Then the biological roles responsible for EZH2 in tongue cancer progression were identified using both pharmacological and siRNA-mediated genetic approaches. Moreover, treatment efficiency of DZNep against tongue cancer was further assessed in the xenograft mouse model.

## RESULTS

### EZH2 is overexpressed in TSCC cell lines and clinical specimens

**Fig 1:**
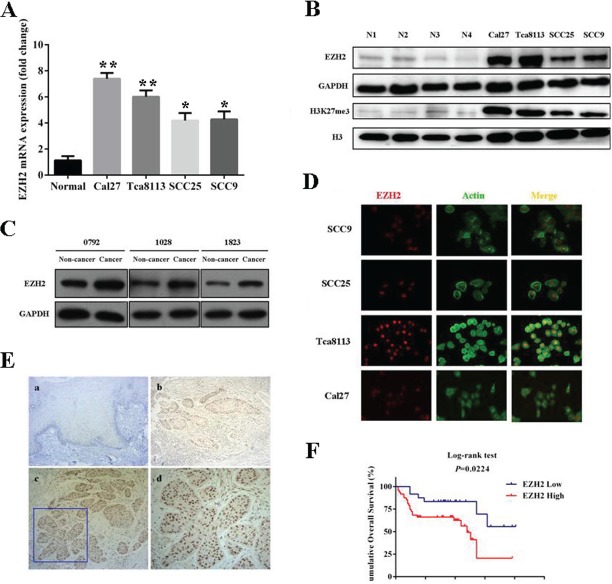
Overexpression of EZH2 and its prognostic significance in TSCC A: EZH2 mRNA levels were determined by real-time RT-PCR in four tongue SCC cell lines and normal tongue mucosa. B: EZH2 and its associated H3K27me protein levels were measured by western blotting in TSCC lines and normal tongue mucosa. Representative images of WB are shown. C: EZH2 protein levels were determined by western blotting in three pairs of tongue SCC and adjacent non-tumor tissue samples. D: Localization of EZH2 protein was identified by immunofluorescence staining (EZH2 was predominantly detected in nucleus). A representative immunofluorescence image is shown. E: EZH2 expression in human tongue SCC specimens was determined by immunohistochemistrical staining. (a: negative expression in normal tongue mucosa(×200); b: low expression in tongue SCC(×200); c: high expression in tongue SCC(×200); d: high magnification of EZH2 high expression(×400)). F: Overall survival analyses of patients with high or low expression of EZH2 were estimated by Kaplan-Meier method and compared with log-rank test. Data showed here are mean ± SD from three independent experiments, *p<0.05,**p<0.01, Student's t-test.

To explore the EZH2 expression in TSCC, we first evaluated both mRNA and protein levels of EZH2 in a panel of TSCC cell lines as compared with normal tongue mucosa. As shown in Fig 1A, EZH2 mRNA levels in all cancerous cells were significantly higher than that in normal mucosa as assessed by real-time RT-PCR assay. The amounts of EZH2 mRNA in Cal27, Tca8113, SCC9 and SCC25 were elevated approximately 7.42, 6.01, 4.27 and 4.18 folds (P<0.01), respectively. The following results derived from western blotting experiments further confirmed the upregulated EZH2 expression in all TSCC cells lines (Fig 1B). The protein levels of EZH2 in Tca8113, Cal27, SCC9 and SCC25 were dramatically increased (P<0.01) when compared with normal tongue mucosa. In addition, H3K27me3 as the primary marker for EZH2 enzyme activity was also quantified in these cell lines. As expected, the expression levels of H3K27me3 in the tongue cancer cell lines was markedly upregulated relative to normal mucosa (Fig.1B).

To further examine EZH2 expression in clinical specimens, we next compared its abundance among three pairs of tongue cancer and adjacent non-tumorigenic tissue. As indicated in Fig.1C, EZH2 protein was remarkably upregulated in tongue cancers as compared to adjacent non-tumorigenic tissue. Then, the expression level of EZH2 was further evaluated by immunohistochemical staining in a retrospective cohort of 84 samples from primary TSCC patients. In accordance with our immunohistochemistry scoring method, EZH2 expression in TSCC and normal tongue mucosa was shown in Table 1. EZH2 levels in all specimens derived from TSCC patients were graded as low (30) or high expression group (54), while its expression levels in normal counterparts were divided into negative (4), low (10) group and high (2), indicating aberrant overexpression of EZH2 in a fraction of TSCC samples (P<0.01). A representative labeling of EZH2 in TSCC and normal tongue mucosa was shown in Fig.1E. In cancer cells, high EZH2 expression was readily identified mainly in nucleus but rarely in cytoplasm. To further characterize the subcellular distribution of EZH2 in tongue cancer, cellular immunofluorescence was performed in tongue cancer cell lines. As shown in Fig.1D, EZH2 was readily detected and identified in nucleus in cancer cell lines examined. Collectively, these data indicate that EZH2 is aberrantly overexpressed in a significant fraction of tongue cancers.

**Table 1:** EZH2 expression in normal tongue mucosa and TSCC

	EZH2 expression			p-values
	Negative	Low	High	
Normal tongue mucosa	4	10	2	< 0.0001
TSCC	0	30	54	

### Overexpression of EZH2 is associated with pathological grade, cervical nodes metastasis and Ki-67 expression and poor prognosis of TSCC

To further understand the significance of EZH2 overexpression in tongue cancers, we set out to identify the potential associations between EZH2 expression and patients' clinicopathological features. Several clinicopathological features (age, gender, pathological grade, tumor size, clinical stage, cervical node status, local invasion) of these patients were summarized in Table 2. In brief, 46 male and 38 female patients were enrolled with mean age 56.1 years (26-76 years). The follow-up period ranged from 2 months to 101 months with average 40.2 months. The detailed relationships between EZH2 expression status and clinicopathological variables of 84 patients were shown in Table 2. There were no significant correlations between EZH2 expression with patients' age, gender, tumor size, local invasion and clinical stage. Noticeably, significant associations between EZH2 abundance with pathological grade and cervical nodes metastasis were found with P-values 0.0027 and 0.0059, respectively.

**Table 2:** Associations between EZH2 expression and multiple clinicopathological parameters in TSCC

Clinicopathological parameters	Cases	EZH2		p-values
		low	high	
Gender	84			
Male	46	17	29	0.8229
Female	38	13	25	
Age				
≤60	52	17	35	0.4898
>60	32	13	19	
Tumor size				
T1-T2	64	26	38	0.1140
T3-T4	20	4	16	
Pathological Grade				
I	37	20	17	0.0027
II-III	47	10	37	
Cervical metastasis				
N(0)	41	21	20	0.0059
N(+)	43	9	34	
Clinical stage				
I-II	36	15	21	0.3635
III-IV	48	15	33	

Given the facts that Ki-67 is a well-established biomarker for cancer cell proliferation and EZH2 has putative proproliferative role during cancer progression, we then ask whether the positive correlation between EZH2 and Ki-67 existed in TSCC. Ki-67 expression was determined by immunohistochemical staining as EZH2 in the same TSCC specimens. Indeed, as shown in Table 3, EZH2 expression was found to be significantly associated with Ki-67 (P =0.0215) in these patients analyzed.

**Table 3:** Correlation between EZH2 and Ki-67 expression in TSCC

Variable	Number	EZH2		p-values
		Low	High	
Ki-67		30	54	0.0215
Low	38	19	19	
High	46	11	35	

To determine relationship between EZH2 expression and TSCC patients' prognosis, we attempted to evaluate the correlation between EZH2 expression and clinical outcomes. At the time of the last follow-up, 39 of 72 (54.2%) patients were alive and disease-free, 9 (12.5%) patients with recurrence and/or cervical nodal metastases, 24 (33.3%) patients died due to local recurrence, metastases or other unrelated diseases. In a Kaplan-Meier survival analysis, as shown in Fig.1F, high EZH2 expression in TSCC was significantly associated with short overall survival (Log-rank, P=0.0224), suggesting that EZH2 overexpression associated with an adverse prognostic outcomes.

To further assess whether EZH2 expression can be identified as a prognostic predictor for TSCC patients, the univariate and multivariate survival analyses (Cox proportional hazards regression model) were performed. In the univariate survival analysis, cervical nodal metastasis and EZH2 expression significantly associated with overall survival (P=0.007, 0.022, respectively), whereas other clinicopathological variables including Ki-67 expression didn't reach the statistical significance as indicated in Table 4. To rule out confounding factors, multivariate survival analysis was carried out including the relevant covariates. In this Cox regression model, EZH2 expression status was found to be an independent prognostic marker for the overall survival of TSCC patients (P=0.047), together with cervical nodal metastasis (P=0.014).

**Table 4:** Univariate and multivariate survival analyses (proportional hazards method) for patients with TSCC

Variable	Univariate survival analysis			Multivariate survival analysis		
	Hazard ratio	95% CI	p-value	Hazard ratio	95% CI	p-value
Gender (male, female)	1.433	(0.662, 3.102)	0.361	1.573	(0.520, 4.757)	0.423
Age (≤60, >60)	0.747	(0.330, 1.688)	0.483	1.066	(0.428, 2.658)	0.891
Tumor size (T1-T2, T3-T4)	1.527	(0.667, 3.495)	0.317	2.602	(0.626, 10.807)	0.188
Pathological grade (I, II-III)	2.201	(1.022, 4.739)	0.044	1.170	(0.462, 2.964)	0.741
Local invasion (No, Yes)	1.306	(0.586, 2.913)	0.514	1.267	(0.453, 3.544)	0.652
Cervical nodal metastasis (N0, N+)	2.973	(1.343, 6.581)	0.007	4.806	(1.381, 16.727)	0.014
Clinical stage (I-II, III-IV)	1.758	(0.820, 3.771)	0.147	.439	(0.097, 1.989)	0.286
Ki-67 expression (Low, high)	1.904	(0.841, 4.311)	0.122	.978	(0.413, 2.314)	0.959
EZH2 expression (low, high)	10.481	(1.402, 78.334)	0.022	8.538	(1.024, 71.187)	0.047

### DZNep treatment inhibits EZH2 through proteosome-mediated protein degradation

**Fig 2:**
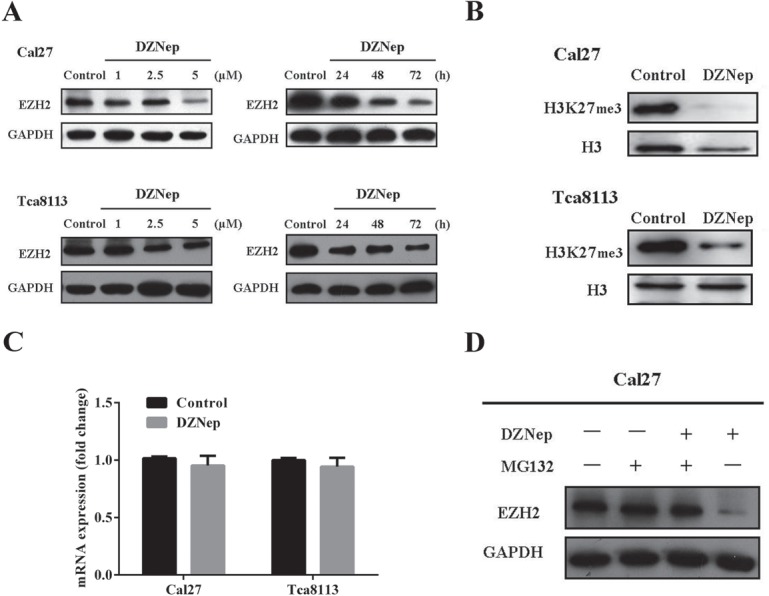
DZNep inhibits endogenous EZH2 by proteosome-mediated protein degradation A: DZNep inhibits EZH2 in a dose-and time-dependent manner in both Cal27 and Tca8113 cells. Representative images of WB are shown. B: H3k27me3 was simultaneously reduced following DZNep exposure (5μM, 72h) in both Cal27 and Tca8113 cells. Representative images of WB are shown. C: Real-time RT-PCR assay for EZH2 mRNA levels following DZNep exposure (5μM, 24h) in both Cal27 and Tca8113 cells. D: EZH2 protein was determined by Western blotting after Cal27 was exposed to DZNep or/and the proteosome inhibitor MG132 for 24h. Representative image of WB were shown. Data showed here are mean ± SD from three independent experiments, Student's t-test.

Previous reports have indicate that DZNep potently depleted the core proteins of Polycomb complex including EZH2 and robustly triggered anti-cancer therapeutic effects in several cancer cells[19, 20]. To determine whether the inhibitory effects of DZNep on EZH2 expression exist in tongue cancer cells, we first measured the EZH2 abundance change following various concentrations and time courses of DZNep treatment. Cal27 and Tca8113 were incubated with various concentrations of DZNep(1, 2.5, 5μM) for varying time courses and then subjected to western blotting assay. As shown in Fig. 2A, DZNep induced a time-dependent and dose-dependent decrease of EZH2 in these two cell lines. The most significant inhibitions were observed when cells were treated with 5 μM DZNep and for 72h. As H3K27 tri-methylation is mainly catalyzed by EZH2 and associated with the PRC2-mediated epigenetic repression in cancer, we then monitored the changes of H3K27 expression after cells treated with DZNep. As expected, the abundance of H3K27me3 was pronouncedly downregulated following DZNep incubation (Fig.2B).

To investigate whether EZH2 reduction by DZNep is due to transcriptional repression, EZH2 mRNA abundance before and after DZNep treatment was compared via real-time RT-PCR. However, EZH2 mRNA remained almost unchanged following DZNep exposure (Fig.2C), suggesting that DZNep might decrease EZH2 via a post-transcriptional way. A line of evidence has indicated the components of PRC2 complex including EZH2 are subjected to proteosome-mediated degradation [20]. Therefore, Cal27 cells were treated with DZNep alone or in combination with proteosome inhibitor MG132 for 24h. Our results indicated that addition of MG132 partially prevented the reduction of EZH2 induced by DZNep (Fig.2D), implying that DZNep inhibited EZH2 probably through increased protein degradation rather than transcriptional repression.

### DZNep inhibits cell growth and migration and invasion, while induces cell apoptosis in tongue cancer cells

**Fig 3:**
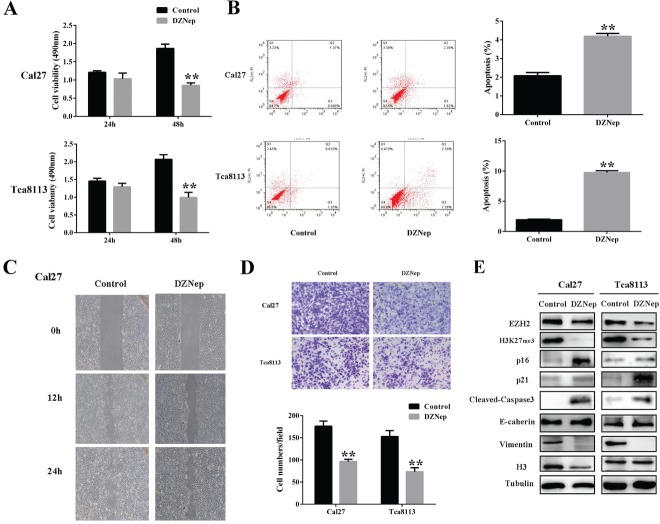
DZNep inhibits cell growth and migration and invasion, while induces cell apoptosis in tongue cancer cells A: Cell proliferation was significantly impaired after DZNep treatment for 48h as measured by MTT assay. B: The percentages of apoptotic cells were remarkably increased after DZNep treatment as determined by flow cytometry. C: Migration potentials of Cal27 treated with DZNep and vehicle were determined by wound healing assay. D: Invasion potentials of Cal27 treated with DZNep and vehicle were determined by modified Boyden chamber assay. E: The protein abundances of several downstream effectors or targets of EZH2 before and after DZNep exposure were measured by Western blotting. Representative images of WB are shown. Data showed here are mean ± SD from three independent experiments, **p<0.01, Student's t-test.

We next set out to clarify the effects of DZNep on cell phenotype and properties in tongue cancer cells. As shown in Fig.3A, DZNep incubation significantly decrease the cell viability at 48h in Cal27 and Tca8113 cell lines, although the inhibitory effects were not much obvious at 24h. Following DZNep treatment (5μM) for 72h, the proportions of cells in G1 phase in Cal27 and Tca8113 were approximately 62.1% and 56.3% respectively, which were much higher than that in cells treated with DMSO (38.6% and 40.2%, P<0.01). Accordingly, the populations of cells in S phase and M phase were significantly decreased upon DNZep exposure in both cell lines. Then, we asked whether these cell-cycle regulators were responsible for DZNep-induced G1 arrest and reduced cell proliferation. Previous studies have indicated that the key cell cycle regulators including p16 and p21 were putative downstream effectors of EZH2 and can be induced in cancer cells [14, 26]. In agreement with these previous studies, our data showed that both p16 and p21 were strongly induced and exhibited remarkable increase following DZNep addition (Fig.3E). Together, these data indicate that DZNep inhibits cell proliferation with robustness at least in part through inducing G1 cell cycle arrest via p16 and p21 upregulation in tongue cancer cells.

The results from cell apoptotic assay indicated a remarkable increase of cell apoptosis in the presence of DZNep. The proportions of apoptotic cells increased from 2.12 to 4.25 % in Cal27 and 1.94% to 9.85% in Tca8113, respectively (Fig.3B). Increased abundance of active cleaved caspase-3 protein further confirmed the apoptosis triggered by DZNep in tongue cancer cells (Fig.3E).

Using wound healing assay and Matrigel invasion assay, the migratory and invasive properties of tongue cancer cells were determined when treated with DZNep. Our results showed that the migration and invasion were both significantly reduced upon DZNep exposure (Fig.3C-D and data not shown for Tca8113). Since E-cadherin has been well established as a master regulator in cancer invasion and metastasis and a key downstream target of EZH2, we then wonder whether E-cadherin induction was responsible for the impaired migration and invasion induced by DZNep. As shown in Fig.3E, DZNep treatment resulted in a pronounced upregulation of E-cadherin protein together with a remarkable decrease of Vimentin in both tongue cancer cells. Taken together, these results suggest that DZNep inhibits cell migration and invasion probably through upregulation of E-cadherin and downregulation of Vimentin.

### DZNep reduces colony formation and CD44+subpoputation in tongue cancer cells

**Fig 4:**
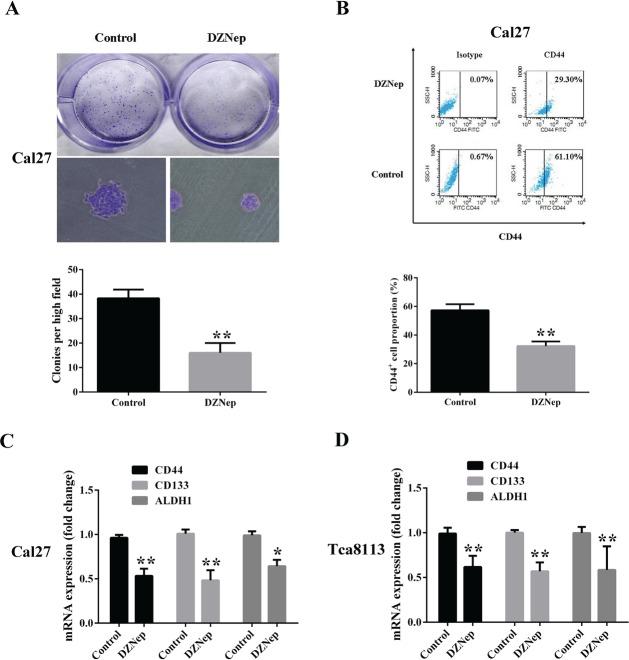
DZNep reduces colony formation and CD44+subpoputation in tongue cancer cells A: The number and size of colonies formed by Cal27 pretreated with DZNep or vehicle as stained by crystal violet. B: The percentage of CD44 positive subpopulation in DZNep or vehicle treated Cal27 cells was determined by FACS. C, D: The mRNA levels of several common markers for cancer stem cell isolation were quantified using real-time RT-PCR assay. Data showed here are mean ± SD from three independent experiments, *p<0.05, **p<0.01, Student's t-test.

Given the essential roles of PRC2 complex in stem cell homeostasis and regulation, we then ask whether DZNep affects the cancer stem cell-like properties in tongue cancer. Firstly, the colony-forming assay was performed to evaluate the effects of DZNep on the colony-forming efficiency which is a surrogate readout of stem cell abundance. The results clearly indicated that the number of colony was much fewer in cells after DZNep treatment than that in cells without DZNep. In addition, the size of colony was also smaller in cells following DZNep treatment (Fig.4A). Then we further sought to measure the fraction of CD44-positive cell population which contained putative cancer stem cells in head neck cancer by fluorescent-activated cell sorting. Notably, the percentage of CD44-positive cell fraction in Cal27 decreased significantly from 60.2% to 30.3% after DZNep treatment as shown in Fig 4C. The similar results were also observed in Tca8113 cells (data not shown). To further confirm the inhibitory effects conferred by DZNep on putative cancer stem cell subpopulation in tongue cancer, the expression levels of several key cell surface makers for cancer stem cell characterization including CD44, CD133 and ALDH1 were compared in the presence or absence of DZNep. Our real-time RT-PCR results indicated that mRNAs of these three makers displayed a significant decrease in cells treated with DZNep in compared with those treated with vehicle only (Fig.4D, E). Taken together, our data suggested that DZNep has a potent inhibitory effect on putative tongue cancer stem cell subpopulation.

### DZNep induces cell senescence and enhanced 5-FU chemosensititvity in tongue cancer cells

**Fig 5:**
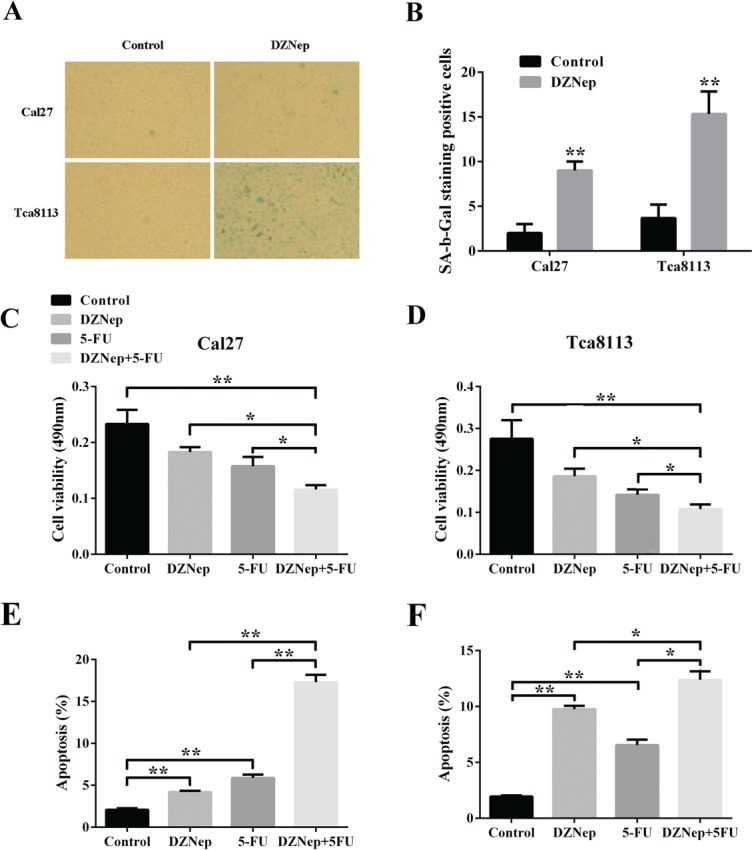
DZNep induces cell senescence and enhanced 5-FU chemosensititvity in tongue cancer cells A, B: SA-β-gal staining positive cells were determined and compared after Ca27 and Tca8113 were treated with DZNep or vehicle for 72h. C, D: Cell proliferation was significantly inhibited by DZNep and 5-FU alone or in combination as assayed by MTT. E, F: The percentages of cell undergoing apoptosis after DZNep or vehicle treatment were measure by flow cytometry and compared accordingly. (Data are mean ± SD from two independent experiments, *p<0.05, **p<0.01, Student's t-test.)

Cellular senescence is one of the common and critical anti-tumor mechanisms underlying cancer therapy and also one aspect of the key biological functions of PRC2 complex [27]. We next sought to clarify whether DZNep was capable to induce cellular senescence in tongue cancer cells. As shown in Fig.5A, DZNep treatment significantly induced cell senescence as evidenced by increased SA-β-gal staining positive cells. Compared with vehicle control (less than 2%), the percentage of SA-β-gal positive cells increased to 14.6% and 8.5% in Tca8113 and Cal27, respectively. Senescent cells undergo growth arrest mediated by two principal pathways including p16/Rb and p53/p21 signaling cascades [28]. After incubation with DZNep, the fraction of tongue cancer cells underwent senescence together with remarkable induction of p16 and p21 (Fig.3E). Collectively, these findings suggest that DZNep triggers cell senescence by activations of both p16/Rb and p53/p21 signaling in tongue cancer cells.

Previous reports have offered some clues that aberrantly overexpression of EZH2 is associated with chemoresistance and its depletion enhanced chemosensitivity in multiple cancers [29]. Here, we asked whether DZNep can enhance anti-tumor activities with the commonly used anticancer drug 5-FU in vitro. Not surprisingly, cell proliferation and viability were significantly impaired when cells were treated with DZNep or 5-FU alone as measured by MTT assay. However, cell viability was more significantly reduced in cells treated with both DZNep and 5-FU in comparison to DZNep or 5-FU alone (Fig.5A, C). Furthermore, the percentage of apoptotic cells was much higher in cells with DZNep and 5-FU incubation than that in cells with single drug incubation as evidenced by flow cytometry (Fig.5B, D). Together, these findings indicate that DZNep has capacity to enhance anti-tumor functions of 5-FU by reducing cell viability and inducing cell apoptosis in tongue cancer cells.

### EZH2 knockdown by siRNA phenocopys the effects of DZNep exposure in tongue cancer cells

**Fig 6:**
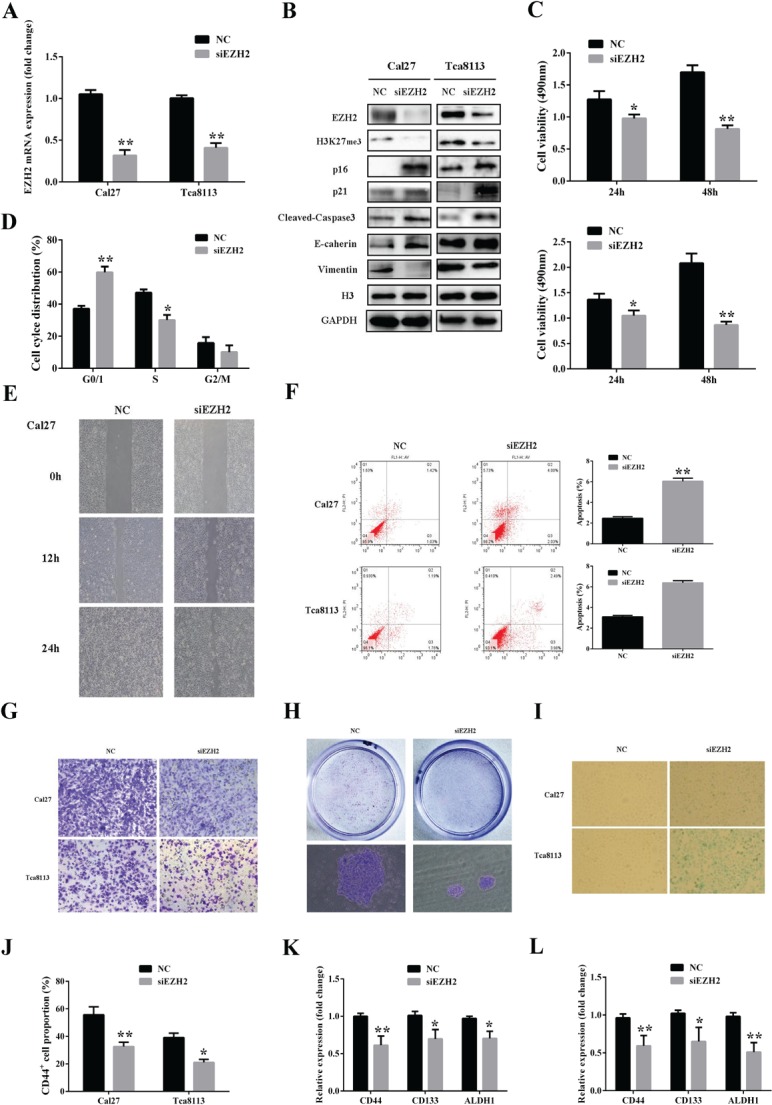
EZH2 knockdown by siRNAs phenocopys the effects of DZNep exposure in tongue cancer cells A: EZH2 mRNA levels after siEZH2 or negative control siRNA transfection in both cell lines as quantified by real-time RT-PCR assay. B: The abundance of EZH2, H3K27me3 and other associated downstream effectors were determined by western blotting following siEZH2 transfection. Representative images of WB are shown. C: Cell proliferation was measured after both cells were transiently transfected with siEZH2 or negative control siRNA. D: Cell cycle distribution of Cal27 followed by siEZH2 and negative control siRNA treatment as measured by flow cytometry. E: Migration potential of Cal27 treated with siEZH2 and negative control siRNA was determined by wound healing assay. F: The percentages of apoptotic cells were remarkably increased after siEZH2 treatment as determined by flow cytometry. G: Invasion potentials of Cal27 and Tca8113 treated with siEZH2 and negative control siRNA were determined by modified Boyden chamber assay. H: The number and size of colonies formed by Cal27 pretreated with siEZH2 or negative control siRNA as stained by crystal violet. I: SA-β-gal staining positive cells were determined and compared after Ca27 treated with siEZH2 or negative control siRNA for 72h. J, K,L: The percentages of CD44 positive subpopulation after Cal27 and Tca8113 transfected with siEZH2 or negative control siRNA for 72h were determined by FACS. The transcriptional level of several common markers for cancer stem cell isolation were quantified using real-time RT-PCR assay. Data are mean ± SD from three independent experiments, *p<0.05, **p<0.01, Student's t-test.

Since DZNep functions as a global potent inhibitors of histone methylation and doesn't act as EZH2 antagonist with high specificity, we further examine that whether these anti-cancer properties exerted by DZNep were attributed to its depletion of EZH2. The siRNA-mediated knockdown strategy was exploited to delineate the phenotype changes after endogenous EZH2 was specifically inhibited in tongue cancer cells. As shown in Fig.6A, B, both EZH2 and H3k27me3 were significantly reduced following the EZH2 siRNAs transfection as compared with non-specific control, thus validating the knockdown efficiency of EZH2 siRNA. Subsequently, the relevant phenotype changes of Cal27 and Tca8113 treated with EZH2 siRNAs were further examined. As displayed in Fig.6C-K, our results indicated that siRNA-mediated EZH2 depletion resulted in impaired cell proliferation, reduced migration and invasion, triggered more apoptotic and senescence cells, less CD44-positive cells and enhanced 5-FU chemosensitivity, largely resembling the results derived from DZNep treatment in vitro. Taken together, these findings strongly suggest that the EZH2 serves as a potent oncogenic regulator involving multiple properties of tongue cancer cells and DZNep executes its anticancer functions, at least in part by its pharmacological inhibition of EZH2 in tongue cancer cells.

### DZNep treatment reduces proliferation, induces apoptosis and inhibits growth of tongue cancer cell in vivo

**Fig 7:**
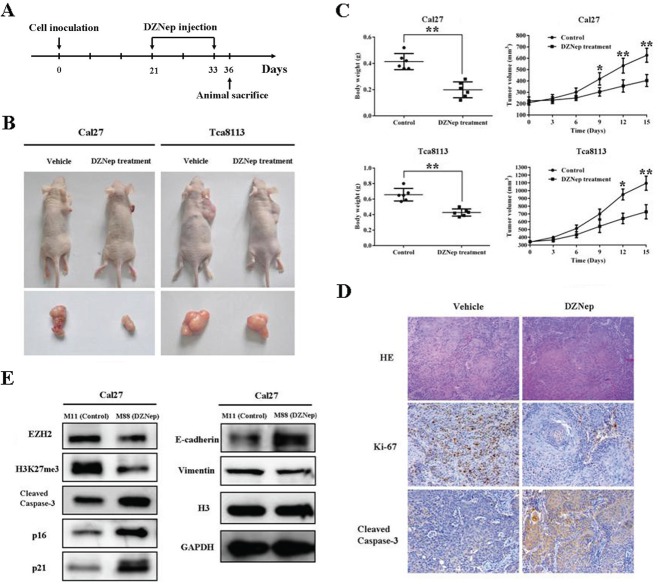
DZNep inhibits tumor growth by inducing intratumoral EZH2 reduction in the TSCC xenograft mouse model A: Time schedule for establishing the TSCC xenograft mouse model and subsequent DZNep administration (2mg/kg body weight, once every 3 days). B: Tumor xenograft samples in mice bearing Cal27 and Tca8113 cells followed by DZNep intraperitoneal injection or vehicle injection. Representative images of mouse in each group are shown (n=6 each group). C: Tumor weights (when sacrificed) and tumor volumes were compared between the mice which received DZNep or vehicle (n=6 each group). D: HE staining, Ki-67 and active caspase-3 immunohistochemical staining in tissue samples derived from mice inoculated with Cal27. E: EZH2 protein and its associated targets were determined by western blotting in samples from mice bearing Cal27 tumors. Data showed here are mean ± SD from three independent experiments, **p<0.01, Student's t-test.

To further testify the therapeutic efficiency of DZNep in vivo, a tongue cancer xenograft model was developed and utilized as described in Fig.7A. After subcutaneous inoculation of Cal27 and Tca8113 three weeks later, tumor masses were readily established and then mice were randomly grouped for drug treatment. These mice were administered DZNep or vehicle by intraperitoneal injection once every three days. Following the first drug injection, tumor masses volumes were monitored and calculated. As shown in Fig.7C, although the tumor growth did not display completely inhibition or recession in mice receiving DZNep, the mean tumor volumes were much smaller in mice treated with DZNep as compared with the mice treated with vehicle only at multiple time points. The final weight of tumor masses were also significantly less in DZNep-treated mice than that in vehicle-treated mice (Fig.7C). During the whole animal experiment, DZNep were well tolerated by mice without obvious abnormalities of behavior, feeding and weight loss during the experiment. We next examined the expression levels of Ki-67 and cleave caspase-3 as makers of cell proliferation and apoptosis by immunohistochemistry in the tumor samples. The immunohistochemical staining showed that in comparison to the control mice, the percentage of Ki-67 positive cancer cells was strongly reduced, whereas the percentage of cleaved caspase-3 positive cells was significantly increased in mice administered DZNep (Fig.7D). To establish the existence of DZNep-induced EZH2 inhibition in vivo, EZH2, H3K27me3 and other potential downstream effectors of interest were further examined in the tumor samples. As indicated in Fig.7E, intratumoral EZH2 and associated H3K27me3 were significantly inhibited after DZNep administration, while several downstream effectors which were normally epigenetically silenced displayed increase with various degrees. Taken together, our findings indicate that intraperitoneally-administrated DZNep effectively exerted its in vivo therapeutic functions probably by inhibition of EZH2 and derepression of EZH2-mediated epigenetic silencing.

## DISCUSSION

Aberrant epigenetic alternations including histone modifications are one of the key hallmarks of human cancer. Many tumor suppressors are epigenetically silenced in this way during cancer development. However, such epigenetic changes are reversible which underscores the possibility that such epigenetic modifications may be ideal targets for cancer therapeutic intervention [30]. In the present study, we provide evidence that EZH2, the key catalysis enzyme for H3K27me3, is abnormally overexpressed in a large fraction of tongue cancers. Its overexpression associates with several aggressive characteristics and serves as an independent prognostic factor for patients with tongue cancer. Moreover, using both pharmacological and genetic approaches, the multiple oncogenic roles of EZH2 and potential underlying mechanisms during tongue progression were identified. Furthermore, the therapeutic efficiency of DZNep against tongue cancer was confirmed in the tongue cancer xenograft model.

EZH2 as the core member of PRC2 has been linked to multiple aggressive cancers including head neck cancers. It usually intertwine with other oncogenic factors and regulators such as Myc, miR-26 and miR-31 to form complex regulatory circuits responsible for cancer development [31, 32]. EZH2 overexpression associated with tumor proliferation, lymph node metastasis, disease stage and poor prognosis of oral squamous cell carcinoma [24, 25]. Moreover, EZH2 has been found to promote malignant transformation of oral leukoplakia, epithelial to mesenchymal transition and cancer stem cell maintenance in head neck cancers [33]. Our data further extended and confirmed the aberrant overexpression patterns of EZH2 in tongue cancers. Briefly, our results showed that EZH2 upregulation was associated with pathological grade, cervical metastasis, Ki-67 expression and reduced overall survival. Therefore, our results together with previous studies indicate that aberrant EZH2 overexpression might be one of the pivotal events during the tongue tumorigenesis and EZH2 might be a novel and critical biomarker for diagnosis and prognostic prediction of patients with tongue cancers.

The clinicopathological significance of EZH2 overexpression in tongue cancers prompt us to further dissect the possible biological roles of EZH2 during cancer progression by loss-of-function assays via both pharmacological and genetic approaches. DZNep has been widely employed to investigate the functions of EZH2 in diverse biological contexts including cancer, largely due to its potency to inhibit the endogenous EZH2 and associated trimethylation of H3K27 in multiple types of cells [20, 21]. Here, our findings indicated that DZNep reduced EZH2 protein abundance in a time-dependent and dose-dependent manner and repressed the H3K27 trimethylation simultaneously. With regard to the potential mechanism behind EZH2 depletion induced by DZNep, our experiments further showed that DZNep exposure induced dramatic reduction of EZH2 probably through protein degradation as evidenced by the facts that there was little change of EZH2 mRNA in the presence of DZNep and remarkable loss of inhibitory effect of DZNep on EZH2 protein in the cotreatment with DZNep and the proteosome inhibitor. These findings have been also supported by previous reports that DZNep triggered the reduction of EZH2 in breast, colorectal and skin cancer cells through increased protein degradation, rather than as a result of transcriptional mechanisms [20, 34]. Furthermore, our findings indicated that DZNep induced not only EZH2 reduction and removal of accompanied H3K27 trimethylation, but also reactivated several known downstream targets including p16 and E-cadherin. Collectively, these results indicate that DZNep is a robust chemical inhibitor against EZH2 through triggering proteosome-mediated protein degradation and has capacity to modulate EZH2-mediated downstream signaling cascade in tongue cancer cell. These findings also suggest that DZNep might be a potent and versatile blocker of EZH2 for cancer therapy irrespective of cancer origin.

Accumulated evidence has established that EZH2 plays pleiotropic tumorigenic roles in cancer progression by regulating a myriad of target genes in multiple cancers [35]. Our loss-of-function studies in vitro indicated that deregulated EZH2 and its associated pathways were involved in diverse aspects of oncogenic phenotype of tongue cancer cells. Previous reports have indicated that EZH2 is identified as a downstream mediator of the retinoblastoma protein (pRB) pathway-E2F pathway which controls multiple key cell-cycle regulators during cell proliferation in normal and cancer cells [36]. Additionally, EZH2 represses the p16, p19 and p15 directly or indirectly which activates the Cyclin D-CDK4/6 complex and promotes progression through G1 phase and cell proliferation [14, 37]. Consistent with these results, our data showed remarkable inhibition of cell proliferation, G1 cell cycle arrest and accompanying upregulation of two key cell-cycle regulators p16 and p21 after EZH2 depletion. Moreover, the closely positive association between EZH2 and Ki-67 expression in clinical samples, reduced Ki-67 staining and tumor growth following EZH2 inhibition in the xenograft model further supported the notion that EZH2 promoted cell proliferation, assumedly by modulating cell cycle in tongue cancer cells.

Previous reports have showed that DZNep selectively induced apoptosis in cancer cells but not in normal cells by preferential reactivation of genes repressed by PRC2 including a novel apoptosis effector FBOX32[20]. Similarly, our data indicated that EZH2 abrogation triggered cell apoptosis and remarkably increased active caspase-3 abundance after DZNep or EZH2-targeting siRNA treatment. More interestingly, we found that EZH2 depletion induced not only cell cycle arrest and apoptosis, but also cell senescence as evidenced by increased senescence-associated β-galactosidase activity. It has been well-established that induction of cell senescence requires the intact pRB-p16 pathway and/or p53-p21 pathway which are interconnected as complementary pathways [28, 38]. In agreement with this notion, EZH2 decrease triggered simultaneous remarkable gains of two senescence-associated regulators p16 and 21. Taken together, these data suggest that EZH2 exerts its oncogenic roles partially through preventing cell apoptosis and senescence and promoting cell proliferation. DZNep has the potent anticancer capacity to simultaneously trigger cell apoptosis and senescence and inhibit cell proliferation as therapeutic advantage against tongue cancer.

Recently, emerging evidence indicates that EZH2 silencing results in inhibitions of cell invasion and migration of several cancer cells and impairs the metastatic spreading in orthotopic xenograft models [23]. Moreover, upregulated EZH2 formed a co-repressor complex with HDAC1/HDAC2/Snail to inhibit E-cadherin which was responsible for the aggressiveness, invasion and metastasis of nasopharyngeal carcinoma cell [39]. Our previous reports together with others have revealed that E-cadherin represents a key master regulator for cancer metastatic spreading in oral cancer cells [23, 40]. Our data indicated that EZH2 downregulation significantly inhibited the invasion and migration properties of tongue cancer cells. Moreover, the key anti-metastatic regulator E-cadherin was significantly upregulated, whereas Vimentin was also downregulated upon EZH2 depletion. Noticeably, the positive correlation between EZH2 overexpression and cervical lymph nodes metastasis further strengthened the finding that EZH2 enhanced cell invasion and migration, probably partially via modulation of E-cadherin in tongue cancers.

Mounting evidence has showed that a limited subpopulation of cancer cells with tumor initiating properties (termed cancer stem cells) largely accounts for cancer initiation, progression and therapeutic resistance [41]. Owing to the key roles of EZH2 as multi-faceted regulator of somatic stem cells and cancer, it's plausible to speculate the potential roles of EZH2 in tongue cancer stem cell maintenance. Chang and his colleagues have showed that increased EZH2 expression in CD44+ /CD24-/low breast tumor initiating cells correlated with the increased percentage of this specific subpopulation. EZH2 promoted expansion of breast tumor initiating cells through RAF1-p-ERK-β-catenin pathway activation [42]. Moreover, phosphorylation EZH2 binds to and methylates STAT3, thereby enhancing STAT3 activity, which promotes tumorigenicity of glioblastoma stem-like cells [43]. Consistent with these findings, our data showed that the proportions of CD44+ cells which harbor putative tongue cancer stem cells in both tongue cancer cell lines were significantly reduced together with several stem cell markers downregulation when endogenous EZH2 was abrogated. These results suggested an important roles of EZH2 in the maintenance of tongue cancer stem cell. This phenomenon was further supported by the others' findings that DZNep predominantly reduced the number and self-renewal capacity of hepatocellular carcinoma initiating cells and selectively abrogated the self-renewal and tumorigenicity of glioblastoma tumor initiating cells [22, 43]. Collectively, our findings suggest that EZH2 plays essential roles in maintenance of tongue cancer cells with stem cell properties. However, further in-depth investigations into the molecular mechanisms behind the EZH2 roles in tongue cancer stem cell homeostasis are warranted.

There is increasing evidence suggests that aberrant overexpression of EZH2 contributes to acquired chemotherapeutic resistance in multiple cancers [44, 45]. EZH2 depletion can efficiently reverse such resistance and sensitized cells to common chemotherapies such as 5-FU and Cisplatin, and produce greater therapeutic efficacy via enhanced cell apoptosis in vitro and in vivo [29, 45]. Based on these previous results, we further determined whether DZNep have the capacity to enhance the 5-FU anti-cancer efficiency in vitro. Indeed, our data indicated that DZNep in combination with 5-FU triggered more pronounced inhibition of cell proliferation and more cell apoptosis when compare with single agent alone. Together with previous findings, it's plausible to speculate that combination of EZH2 inhibitors and standard chemotherapy may have superior treatment efficiency against tongue cancer, although much work is still needed to further dissect the underlying mechanisms and substantiate the therapeutic advantages in animal models and clinical trials.

Given the inherent reversible nature of epigenetic modifications during cancer progression, DZNep has been widely exploited as a potent epigenetic modulator and inhibitor to deplete cellular levels of the PRC2 members including EZH2 and selectively remove histone H3K27 trimethylation mark in multiple cancers, thus exhibiting promising anticancer activity [15, 20]. As expected, our data from DZNep therapy in tongue cancer xenograft model indicated that DZNep delivery efficiently depleted intratumoral EZH2, significantly inhibited the tumor growth by reducing cell proliferation and triggering cell apoptosis. Although previous studies showed DZNep was a global histone methyltransferase inhibitor and not specific for EZH2 inhibition and we can't rule out other unrecognized mechanisms responsible for DZNep-mediated anticancer effects, however, these findings together with others suggested that it yielded its anticancer effects at least in part via disturbing the EZH2 oncogenic pathway irrespective of cancer types[26, 34, 46]. However, it still should be noted that complete abrogation of tumor mass growth and cancer remission in mice weren't observed, whereas tumors continued to grow at a much slower rate following DZNep injection. It is not surprising that it may be due to the unfavorable pharmacokinetics, suboptimal dosages and treatment course of DZNep [47]. To achieve optimized therapeutic outcomes, several strategies has been tried and verified, for example DNZnep in combination with other epigenetic compounds like DNA methyltransferase inhibitors 5'-AzaC and histone deacetylase inhibitors, gene-specific small interference RNA molecules or more specific EZH2 inhibitors. Combined epigenetic therapies with DZNep and histone deacetylase inhibitors have superior efficacy against multiple cancers [19, 26]. Our unpublished data also indicated that DZNep together with panobinostat or sodium butyrate exhibited more potent anticancer efficacies against tongue cancer cell in vitro and in vivo. More importantly, very recently several potent and high selective small-molecule inhibitors of EZH2 have been developed and testified their therapeutic efficiencies in multiple cancers with promising results, thus portending the potential clinical use of EZH2 inhibitors for cancer treatment in future [48, 49]. Taken together, our data provide validation of EZH2 as a potential therapeutic target for tongue cancer treatment and suggest that pharmacological abrogation of EZH2 by native and synthetic chemical compounds might hold great promise as a new therapeutic strategy against cancer.

In conclusion, our results indicate that EZH2 serves as a key oncogenic driver during tongue cancer progression and functions as a novel biomarker for diagnosis and prognostic prediction for patients with tongue cancers. DZNep inhibits EZH2 through inducing protein degradation and reverse PRC2-H3K27me3-mediated gene silencing to yield its anticancer effects. We anticipate that pharmacological and genetic disruption of EZH2 and its signaling cascade may represent a novel therapeutic strategy against tongue cancers.

## MATERIALS AND METHODS

### Tongue cancer cell lines and chemical reagents

Four human TSCC cell lines Tca8113, Cal27, SCC9, SCC25 were used in the present study. Tca8113 was previously established at Shanghai Jiaotong University (a generous gift from Professor Wantao Chen). The other three cells were purchased from American Type Culture Collection (ATCC). Cancerous cell lines were grown in DMEM (Invitrogen) supplemented with 10% FBS (Hyclone) and 100 units/ml penicillin and streptomycin, and maintained in a humidified incubator with 5%CO2 at 370C. DZNep was purchased from Biovision (Category NO. 2060-250) and Cayman (Category NO. 13828) commercially and dissolved in DMSO as stocking solution. Cells were treated with diverse concentration of DZNep for the indicated times. For the DZNep and MG132 co-treatment, the cells were treated with DZNep 12h followed by the addition of MG132 at 5μM for another 12h. For the chemotherapy sensitivity assay in vitro, cells were treated with 5-fluorouracil (Sigma, 2.5mg/ml) or/and DZNep (5μM) for the indicated times.

### EZH2 siRNA synthesis and transfection

Three small interference RNA molecules targeting human EZH2 were synthesized and purchased from Shanghai GenePharma Company. The EZH2-targeting sequences with confirmed efficiency and specificity have been reported before [50-52]. The knockdown efficiency of each siRNA was further confirmed by in vitro delivery to cells in our prior experiments. To enhance the silencing efficiency and reduce potential off-target effects, a pool mixture of equal quantities of EZH2 siRNAs (100nM total, 33.3nM each) was employed to deplete the endogenous EZH2 in cancer cell lines using lipofectamine 2000 (Invitrogen) according to the manufacturer's recommendations. After siRNA transfection for 48h or 72h, the cells were harvested for the further experiments.

### Cell immunofluorescence and imaging

The cancerous cells were seeded and grown on glass coverslips 24h prior to experiment. After fixed with 4% paraformaldehyde-PBS for 15 min, cells were then permeabilized in 0.1% Triton X-100 in PBS and sequentially blocked with 3% bovine serum albumin for 30 min. Following the overnight incubation with primary antibody specific for EZH2 (Cell signaling, #5246, 1:200 dilution), these cells were further incubated with appropriate secondary antibodies and cytoskeleton actin staining. Immunofluorescence was visualized under a Zeiss fluorescence microscope and image-captured.

### RNA extraction and Real time RT-PCR

Total RNA was extracted from cells with 80% confluence using Trizol reagent (Invitrogen). RNA was reversely transcribed into first strand cDNA using PrimeScriptTM RT reagent kit (Takara). The generated cDNA was used as template for real-time PCR reaction using SYBR Premix Ex TaqTM kit (Takara) following the supplier's instructions. The gene-specific primers for human EZH2, CD44, CD133, ALDH1 and GAPDH were purchased commercially (Invitrogen and Qiagen). Relative mRNA expression of each gene as compared to internal control GAPDH was quantified using comparative CT method.

### Western blotting analysis

The cells with 80% confluence in 25 cm2 culture flasks were lysed in ice-cold buffer containing protease inhibitor cocktail (Roche). Equal amounts of protein samples were loaded and separated by 12% SDS-PAGE and transferred to PVDF membranes (Millipore) which then blocked by 5% non-fat dry milk. These blots were incubated at 40C overnight with primary antibodies EZH2, cleaved Caspase-3, H3K27me3, H3 (1:1000 dilution) from Cell signaling, E-cadherin (BD Biosciences, 1:2000 dilution), Vimentin (Santa Cruz, 1:1000 dilution), p16 (Santa Cruz, 1:500 dilution), p21 (Santa Cruz, 1:500 dilution), GAPDH (Santa Cruz, 1:2000 dilution) and tubulin (Sigma, 1:3000 dilution) followed by incubations with secondary antibodies. The relative levels of each protein were quantified with Quantity One software (Bio-rad) and GAPDH/tubulin or H3 served as control.

### MTT assay

Cell proliferation and viability were monitored by absorbance using the MTT assay. Approximately 1000-3000 cells/well were seeded in the 96-well plates. At the indicated time-points, 5mg/ml MTT (Sigma) was added to the cells and incubated at 370C for another 4h. Absorbance at 490 nm was measured with an automatic enzyme-linked immunosorbent assay reader (BioTek Instruments).

### Flow cytometry analysis

Cells were treated with trypsin and resuspended as single-cell suspension. For cell cycle analysis, cells were fixed in 70% ethanol and then stained with propidium iodide following RNase treatment. The stained cells were further analyzed for DNA content and cell cycle distributions using CellQuest software. For apoptosis assay, cells were stained with Annexin V:PE Apoptosis Detection Kit (BD Bioscience). For cell surface maker assay, cells were labeled with human fluorochrome conjugated anti-CD44-APC (BD Pharmingen) and sorted by BD FACSVantage flow cytometer. The corresponding immunoglobulins conjugated APC was used as isotype controls in each experiment.

### In vitro cell invasion and wound healing assay

Cell invasion assay was performed using Matrigel invasion chambers (8-μm pore size, Corning) in 24-well plates. Cell inserts were pre-coated with 100% Matrigel (BD Pharmingen) 24h prior to experiment. Forty-eight hours after siRNA transfection or drug treatments, cells were seeded into the upper chambers with medium containing 1% FBS. Complete medium containing 10% FBS in the lower chambers served as chemoattractant. The non-invading cells were gently removed with a cotton swab and those invasive cell located on the lower side were stained with crystal violet, photographed and counted. For wound healing assay, cells were grown into monolayer and scratched using a sterile 200μL pipette. Cell migration was observed at various time-points later by microscopy. Images of 10 scratches per cell line were captured at the same locations during the experiment and then compared with Image J software.

### Senescence β-galactosidase cell staining

Senescence β-galactosidase cell staining was performed using staining kit purchased from Cell signaling (#9860) and performed accordingly. Briefly, cells were fixed in 2%formaldehyde/0.2%glutaraldehyde/PBS for 15 minutes at room temperature and stained using β-galactosidase staining solution at 370C overnight. The percentage of SA-β-gal positive cells was calculated from ten randomly chosen fields. At least 500 cells were analyzed per experiment.

### Colony forming assay

One thousand cells pretreated with DZNep (5μM, 48h) or vehicle were placed into 6-well plates or dishes and allowed to grow for two weeks. The cells were then fixed and stained with crystal violet. The colonies were further visualized under an invert microscope and photographed. Cell aggregations with more than 50 cells were defined as colonies and then counted.

### TSCC xenograft model and *in vivo* DZNep administration

All the animal protocols in this study were in accordance with the institutional animal welfare guideline of Nanjing Medical University. Six-week old male nu/nu mice were injected subcutaneously on the right flank with 2×106 Cal27 or Tca8113 cells respectively. Three weeks later, these mice (6 mice per group) bearing tumors were randomly divided into two groups which were scheduled to receive the following treatments: 2mg/kg DZNep, once every three days by intraperitoneal injection for consecutive two weeks or vehicle only. The DZNep stockings were diluted in PBS immediately before use. The animal experiments were terminated three days after the last injection. The tumor diameters were measured by calipers every 3 days when tumor masses were identified. Tumor volume was calculated as follows: volume=a×b2/2. The a was defined as the longest diameter, whereas the b as the shortest diameter. Tumor weights were also measured upon tumor samples were harvested.

### Patients and tissue specimens

A total number of 84 patients with primary TSCC (2001 Jan.-2010 Dec.) receiving surgical treatment at the Department of oral and maxillofacial surgery, Nanjing Medical University were enrolled. Patient inclusion criteria were described as follows: (1) primary tongue squamous cell carcinomas without any prior history of chemotherapy or radiotherapy; (2) patients underwent radical tumor resection and neck lymph node dissection (elective or therapeutic neck dissection as required); (3) detailed clinical, pathological and follow-up data (follow-up data available for 72 patients). The archived tissue samples were retrieved and haematoxylin-eosin stained slides of each patient were further analyzed to confirm the previous histological diagnosis according to the established histological criteria. Sixteen samples of normal tongue mucosa were obtained from other non-cancer surgeries during the same period. The normal morphologic features were confirmed under the microscope for these normal mucosa. All these patients gave written informed consent in accordance with our institutional guidelines. This study protocol was reviewed and approved by the Research Ethic Committee of Nanjing Medical University.

### Histopathological evaluation and immunohistochemistry

Immunohistochemical staining for EZH2, Ki-67, active caspase-3 was performed similarly as our previous reports [40, 53]. The immunoreactivity in each slide was evaluated independently by two senior oral pathologists without knowledge about the clinical and pathological data. Negative controls (without primary antibody incubation) were included in each staining run. Immunoreactivity was semi-quantitatively evaluated on the basis of staining intensity and distribution using the immunoreactive score which was calculated as intensity score × proportion score as we reported previously [40, 53]. The immunoreactivity of each slide was divided into three groups based on the final score: 0, negative; 1-4, low expression; 4-12, high expression.

### Statistical analysis

All quantitative data in the present study was shown as mean ± SD unless otherwise stated. Statistical comparisons were performed by Student's t-test or ANOVA as appropriate. For immunohistochemical analyses, the associations between EZH2/Ki-67 expression and various clinicopathological parameters were evaluated using Fisher exact test or χ2-test as indicated. The overall survival rate was estimated using Kaplan-Meier method and compared with log-rank test. The prognostic analyses were performed by univariate and multivariate Cox regression models to determine the individual clinicopathological variables with overall survival. P values less than 0.05 (two-sided) were considered statistically significant. All statistical analyses were performed using Graphpad Prism5 or SPSS 18.0.
